# Avoidance of harvesting and sampling artefacts in hydraulic analyses: a protocol tested on *Malus domestica*

**DOI:** 10.1093/treephys/tpv130

**Published:** 2015-12-22

**Authors:** Barbara Beikircher, Stefan Mayr

**Affiliations:** 1 Institute of Botany, University of Innsbruck, Sternwartestraße 15, 6020 Innsbruck, Austria

**Keywords:** embolism, hydraulics, plant harvest, refilling

## Abstract

A prerequisite for reliable hydraulic measurements is an accurate collection of the plant material. Thereby, the native hydraulic state of the sample has to be preserved during harvesting (i.e., cutting the plant or plant parts) and preparation (i.e., excising the target section). This is particularly difficult when harvesting has to be done under transpiring conditions. In this article, we present a harvesting and sampling protocol designed for hydraulic measurements on *Malus domestica* Borkh. and checked for possible sampling artefacts. To test for artefacts, we analysed the percentage loss of hydraulic conductivity, maximum specific conductivity and water contents of bark and wood of branches, taking into account conduit length, time of day of harvesting, different shoot ages and seasonal effects. Our results prove that use of appropriate protocols can avoid artefactual embolization or refilling even when the xylem is under tension at harvest. The presented protocol was developed for *Malus* but may also be applied for other angiosperms with similar anatomy and refilling characteristics.

## Introduction

Plant hydraulics, in particular embolism formation and reversal, has become an important issue in plant physiology (Sperry 2013). Long distance water transport in the xylem conduits depends on hydraulically continuous water columns as it occurs under tension and in a metastable state (e.g., Tyree and Zimmermann 2002). Drought and/or freeze–thaw events can break these columns (cavitation) resulting in air-filled conduits (embolism), which block the water transport and may eventually lead to damage or even dieback of the plant (Tyree and Sperry 1988, Choat et al. 2012). Over the past decades, numerous studies have dealt with various aspects of plant hydraulics such as hydraulic efficiency, vulnerability to embolism, native embolism and refilling. However, due to the particular biophysical characteristics of water transport in plants (Tyree et al. 1994), studies on xylem functionality are complicated (Sperry 2013). Recent studies have revealed that not only should measurement techniques be scrutinized (e.g., Melcher et al. 2012, Cochard et al. 2013) but also the very first step of measurements, i.e., harvesting and sampling of the plant material, has become a central question of issue (Wheeler et al. 2013, Trifilo et al. 2014, Torres-Ruiz et al. 2015, Venturas et al. 2015).

Collecting plant material for destructive hydraulic measurements includes (i) cutting the whole plant or plant parts (in the following referred to as ‘harvesting’) and (ii) excising the target section (sample) actually used for hydraulic measurements out of the harvested plant or plant part, respectively, and preparing it for measurements (in the following referred to as ‘sampling’). For studies which require that the native hydraulic state in the plant is preserved, e.g., analysis of native embolism, both steps have to be carried out carefully to avoid an artefactual increase (artefactual embolization; e.g., Wheeler et al. 2013) or decrease (refilling; e.g., Trifilo et al. 2014) in loss of hydraulic conductivity (PLC). For practical reasons, harvesting often has to be done during the day when native tension in the plant is high due to transpiration. This can cause problems when air is sucked into the cut, and probably also neighbouring conduits (see Melcher et al. 2012), leading to artefactual embolization. Therefore, even mid-range values of xylem tension, which, for example, occur during midday under transpiration, are critical. This problem can be reduced but not avoided by harvesting under water. However, with woody plants, this often is not possible.

The consecutive sampling should in any case be done under water, whereby the required minimum sample length depends on the measurement technique used in the following (see Melcher et al. 2012). Already Dixon (1914) and several decades later Zimmermann (1978) pointed to possible artefacts during sampling (Wheeler et al. 2013, Sperry 2013). Nevertheless, precautionary protocols were maybe not always given the necessary attention over the following years until the study of Wheeler et al. (2013). These authors showed that sampling while the xylem is still under tension can significantly reduce hydraulic conductivity even when the cuts are made under water. They assumed that during conduit severing, microbubbles are drawn into open conduits resulting in an artefactual blockage or even cavitation of the conduit. This finding has meanwhile also been proofed by image analyses by Torres-Ruiz et al. (2015). To avoid a possible artefact, it was suggested to relax the xylem tension prior to sampling. This may be done either by allowing the harvested plant to rehydrate for a given time with its basal end kept in deionized water while sealed into a plastic bag or a special sampling design for rapid relaxation (see Wheeler et al. 2013). This procedure, though, is not without controversy. In a follow-up study, Trifilo et al. (2014) reported that xylem rehydration prior to sampling can induce refilling. The authors advise girdling the phloem prior to harvesting and tension relaxation as the phloem probably is responsible for refilling. Also Venturas et al. (2015) found some evidence for refilling upon rehydration while they did not observe a tension-cutting artefact in their study species. According to Torres-Ruiz et al. (2015), embolism induced by cutting depends on the species, the xylem tension and the degree of native embolism at the moment of sampling. Thereby xylem anatomy and refilling ability may be decisive, whether a special sampling protocol is required for certain hydraulic measurements on a given species.

When we commenced a new project, which aimed at following annual courses of native embolism in *Malus domestica* Borkh., various test measurements were carried out to develop an adequate harvesting and sampling protocol. With this protocol, possible artefacts should be avoided even when the first cut is made in air under transpiring conditions. The protocol was tested for the risk of artefacts (or their successful avoidance), considering conduit length, time of day of harvesting, shoot age and seasonal effects. The outcome of this study corroborates the findings of other authors that hydraulic analyses can be biased by artefacts. We herewith present an elaborated protocol, which we deem appropriate for harvesting and sampling in *M. domestica* and possibly also many other tree species.

## Materials and methods

### Plant material and experimental design

The experiment was carried out in commercial apple orchards in northern Italy on different apple cultivars. *Malus domestica* Borkh. var. ‘Braeburn’, ‘Gala’, ‘Golden Delicious’, ‘Nicoter’ and ‘Red Delicious’ were grown in orchards surrounding Latsch (South Tyrol, 643 m above sea level (a.s.l.); 46°37′N, 10°52′E) and the cultivar ‘Pinova’ around the neighbouring village of Tarsch (837 m a.s.l.; 46°36′N, 10°53′E). (For details regarding climate, age and tree height, see Beikircher and Mayr 2013, Beikircher et al. 2013.)

Tests on the impact on length of harvested branches and xylem relaxation prior to sampling were carried out in September 2010 on Golden Delicious on branches harvested during midday. For the comparison of hydraulic parameters of samples collected under high (day) and low (predawn) native tension, branches were collected in May 2011 in the afternoon (between 12:00 and 14:00 CET) and before dawn of the following morning (between 04:00 and 05:00 CET). Measurements were done on previous-year shoots of all cultivars, and for Braeburn, Golden and Red Delicious also on current-year shoots. At that time, current-year shoots had just started to develop and were 10 cm long at a maximum. In July 2011, a second set of branches was collected in the afternoon (between 12:00 and 14:00 CET) and measurements carried out on previous- and current-year shoots.

### Harvesting and sampling of branches

Measurements were made on 10 trees per cultivar randomly chosen in the orchards. For the experiments in May and July 2011, water potentials were measured on three leaves per tree with a pressure chamber (Model 1000 Pressure Chamber, PMS Instrument Company, Corvallis, OR, USA). Then, ∼100 cm long, west-exposed branches at breast height were cut at the base. This first cut was made in air and branches were then immediately recut under water three to five times through the first annual node. Branches were then wrapped in dark plastic bags with wet paper towels and left with their basal end in water for ∼30 min to rehydrate. Afterwards, branches were taken out of the water, tightly wrapped in the plastic bags and transported to the laboratory. There, branches were kept in a cold temperature chamber (5 °C) until sampling and hydraulic measurements on the following day.

For sampling, branches were first cut back up to five times under water from their basal end up to the previous-year shoot section. Then, two samples, ∼40 mm long, were cut from the centre of the respective shoot (previous-year, current-year). Therefore, the shoot was recut under water repeatedly in centimetre-steps at both sides alternatively up to the final length, as suggested by Zimmermann (1978). Finally, each sample end was trimmed under water five to seven times with a sharp wood-carving knife. Thereby, to obtain a smooth cut surface and avoid squeezing of conduits, thin slices <0.4 mm thick were cut off. Also, trimming was done at both sides alternatively.

**Figure 1.**
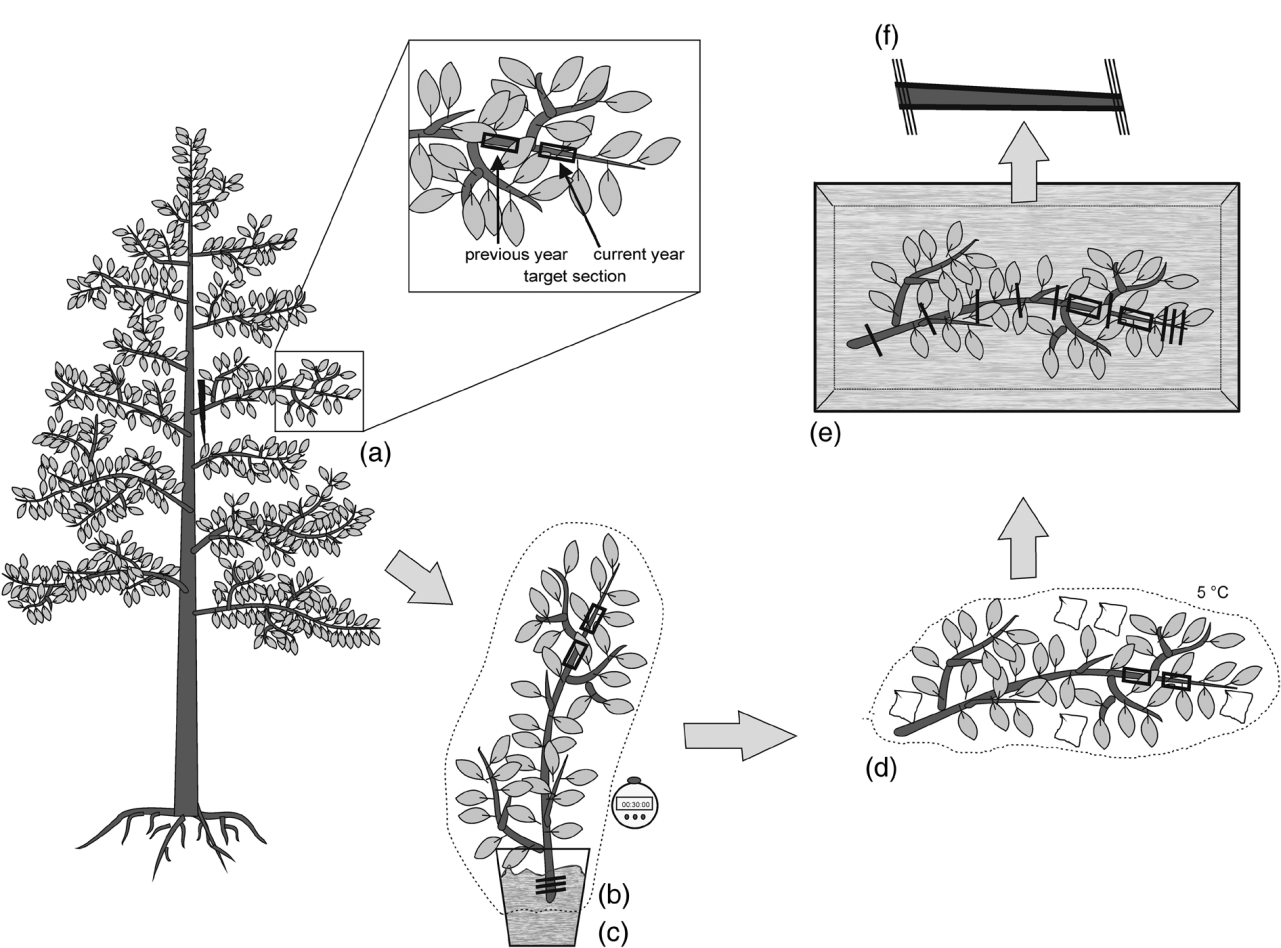
Harvesting and sampling protocol to avoid artificial changes in native embolism levels in target sections used for hydraulic measurements. (a) Cut branch exceeding maximum conduit length several folds. (b) Cut branch back under water several times. (c) Cover with black nylon bags and rehydrate for 30 min. (d) For transportation and storage, wrap branch tightly in black nylon bags together with wet paper towels. Store at 5 °C. (e) Excise target segment by cutting branch several times from both sides alternatively under water. (f) Trim sample ends with a sharp wood-carving knife.

To test for the influence of harvested branch length on artefactual embolization, 40- and 100-cm-long branches of Golden Delicious were harvested in air and under water, respectively. After rehydration, sampling was done as described above. Additionally, 100-cm-long branches were harvested under water and immediately afterwards leaves were removed under water with a sharp razor blade. For hydraulic measurements, samples were then cut from the basal and the distal part of the current-year shoot. For an overview of the harvesting and sampling protocol, see Figure 1.

### Measurements of native embolism and water content

For the measurement of native embolism, samples were debarked prior to the trimming as described above and sealed under water in tubes connected to a ‘xylem hydraulic conductance and embolism measurement system’ (Bronkhorst, Montigny les Cormeilles, France). The level of native embolism was expressed as percentage loss of hydraulic conductance (PLC) and measured as the hydraulic conductance at 4.5 kPa before (*k_i_*) and after (*k*_max_) removal of embolism by repeated high pressure flushes at 95 kPa for 20 min (Sperry et al. 1988; Eq. (1))

(1) $$\mathrm{PLC}=100-\left(\frac{k_{i}}{k_{\max}}\times 100\right)$$

To calculate maximum specific conductivity (*k*_s_), the maximum flow rate of samples obtained after flushing was normalized by sapwood cross-sectional area, sample length and pressure (see Beikircher and Mayr 2008, Beikircher et al. 2013).

For water content analysis, samples were also debarked and the fresh weights (FW) of bark and wood were measured with an analytical balance (Sortorius BP61S, 0.0001 g precision, Sartorius AG, Göttingen, Germany). After oven drying at 80 °C for 48 h, dry weight (DW) was determined and the water content expressed as percentage of DW (WC_%DW_; Eq. (2)).

(2) $$WC_{\%\mathrm{DW}}=\frac{\mathrm{FW}-\mathrm{DW}}{\mathrm{DW}}\times 100$$

### Maximum conduit length

For the analyses of maximum conduit length, branches of Braeburn, Golden Delicious and Red Delicious were connected to an air pump (Eheim 400, Eheim GmbH, Deizisau, Germany). While air pressure (40 kPa) was applied at the basal end, the distal end was repeatedly shortened by ∼0.5 mm under water. Branches were recut until a stream of air bubbles indicated that a conduit had been cut open on both sides (Ewers and Fisher 1989, Nolf et al. 2014). Remaining length was assumed as maximum conduit length.

### Number of samples and statistics

For each set of measurement, 10 branches were harvested (one per tree) and out of each branch, samples for hydraulic measurements were cut from previous-year and/or current-year shoots, respectively. Differences between basal and distal samples within shoots, time of harvesting (afternoon, predawn) in May, differences in shoot age (previous year, current year) in July and differences between PLC values measured in May and July for each shoot age were tested with the Student's *t*-test (normal distribution and equal variances) or the non-parametric Mann–Whitney *U*-test (normal distribution and unequal variances). Differences within cultivars in May, including afternoon and predawn harvesting as well as different shoot ages, were tested with an analysis of variance using the Bonferroni test (equal variances) or the Tamhane test (unequal variances). All tests were made at a probability level of 5% using SPSS (IBM SPSS Statistics, version 21).

## Results and discussion

Harvesting and sampling plant material are critical steps in hydraulic analyses, especially when the native hydraulic state of the sample has to be preserved. This proves to be even more difficult when the first cut (i.e., harvesting) has to be made under transpiring conditions and in air. We developed a harvesting and sampling protocol we deem appropriate to ensure correct measurements of native embolism and hydraulic conductivity in *M. domestica*. Our protocol stipulates harvesting branches exceeding maximum conduit length several folds (Figure 1a). The basal ends of harvested branches are then immediately immersed in water and recut several times (Figure 1b). Branches are rehydrated for ∼30 min under dark plastic bags to allow xylem tension in the harvested branches to equilibrate (Figure 1c). Branches are wrapped tightly in dark nylon bags together with wet paper towels for transport to the laboratory and stored at 5 °C (Figure 1d). Sampling is then done on relaxed branches by cutting several times under water alternating from both sides down to the target section (Figure 1e) and finally carefully trimming both ends with a sharp wood-carving knife (Figure 1f; for details, see Materials and methods). This protocol was designed based on the findings of various test measurements to exclude both an artefactual increase and a decrease in PLC.

**Figure 2.**
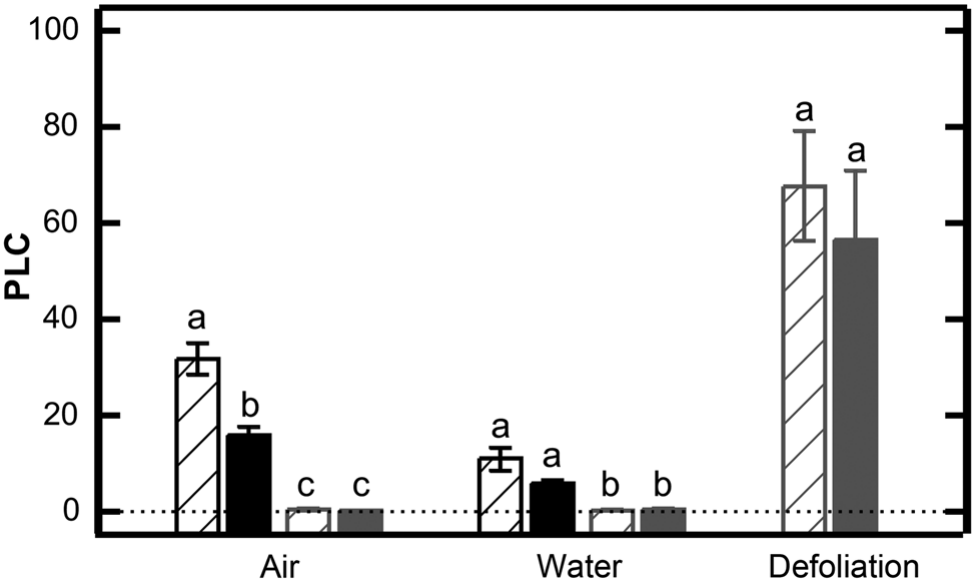
Percentage loss of hydraulic conductivity in basal (hatched bars) and distal (solid bars) of current-year shoots of *M. domestica* var. ‘Golden Delicious’. Harvested branches were 40 cm (black) or 100 cm (grey) long. Harvesting was done in air and under water, respectively, and branches then rehydrated for 30 min prior to sampling. For the defoliation experiment, branches were harvested in air and immediately afterwards leaves were removed under water. Different letters indicate significant differences (*P* < 0.05) between branch length and sample position within a harvesting approach (i.e., harvesting in air, under water or defoliation). Please note that for better visualization, the zeropoint of the *y*-axis (dotted line) is offset.

One important strategy to avoid artefactual embolism is to ensure a sufficient distance between the first (harvesting) cut and the target section used for hydraulic measurements (Melcher et al. 2012, Torres-Ruiz et al. 2015). Maximum conduit length in our study cultivars ranged from 30.5 ± 3.04 cm (Golden Delicious) to 31.27 ± 3.26 cm (Braeburn). However, as branches were highly branched and crooked (see Beikircher et al. 2013), average conduit length may probably be rather in the range of 8 cm as reported by Cohen et al. (2003) for various apple cultivars. When harvested branches were shorter than 1.5 maximum conduit length, PLC was significantly higher in basal samples of current-year shoots compared with distal ones. In branches exceeding maximum conduit length at least threefold, no difference was observed (Figure 2). This points to air entry in conduits during harvesting in air. Harvesting short branches under water reduced but did not completely avoid artefactual embolization (Figure 2). In basal samples of short branches harvested in water, loss of conductivity was significantly lower (11%) than in basal samples of short branches harvested in air (31%). However, PLC was still significantly higher than in long branches (0.2%; Figure 2). As branches were under tension during harvesting due to transpiration, this was most likely related to microbubbles developing during cutting, which proceeded to the target section, similar to the sampling artefact as described by Wheeler et al. (2013). In longer branches, bubbles did not reach the conduits of the target section. These findings, thus, strongly underline the importance of length of harvested branches regardless of whether harvesting in air or under water.

**Figure 3.**
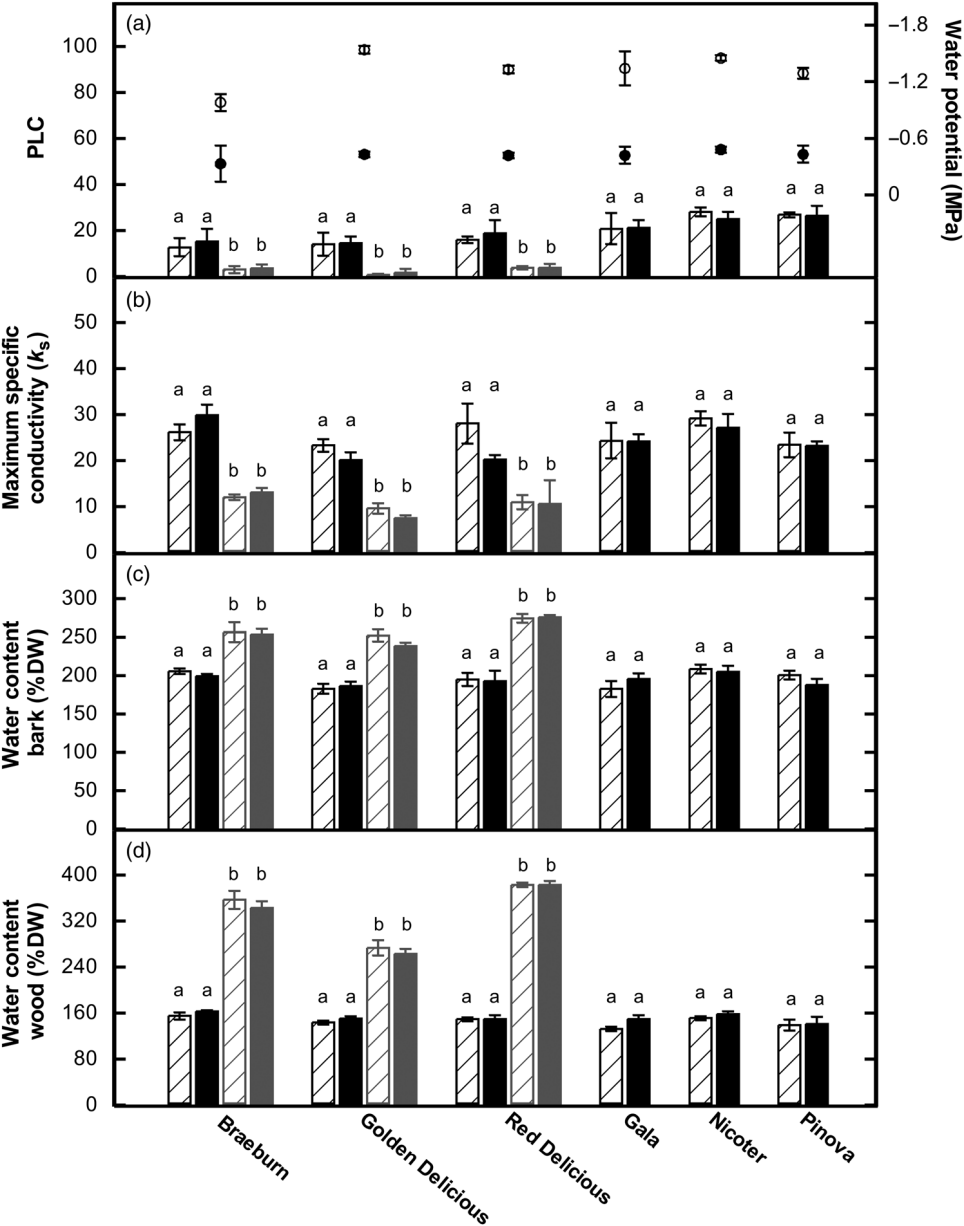
(a) Percentage loss of hydraulic conductivity (PLC, bars) and leaf water potential (circles), (b) maximum specific conductivity, (c) water content of bark and (d) wood of six different cultivars of *M. domestica*. Measurements were made on previous-year (black) shoots of branches harvested in May 2011 in the afternoon (hatched bars, open circles) or before dawn (solid bars, filled circles), respectively. In the cultivars Braeburn, Golden and Red Delicious, also current-year shoots (grey bars) were analysed. Letters show significant differences between afternoon and predawn values within the respective shoot age and cultivar. Means ± SE.

According to the findings of Wheeler et al. (2013) and Torres-Ruiz et al. (2015), even with the first cut far away from the target region, artefactual increase in PLC can occur when sampling is done immediately upon harvesting while the xylem is still under tension. Indeed, when branches were harvested under water and all leaves removed immediately, i.e., without prior relaxation, high embolism levels were observed in the basal as well as in the distal part of current-year shoots (Figure 2). Although long branches were used for this test experiment, PLC was more than twice that of short branches cut in air (see above; Figure 2). This indicates that, by cutting leaves off under tension, numerous entering points for air were created. A reduced tension, thus, should help to avoid artificial embolism, and in our protocol, we therefore included a rehydration step. To test whether this procedure was sufficient to avoid a tension-cutting artefact during sampling, we harvested branches before dawn as well as during midday. Predawn mean leaf water potential prior to harvesting was less than −0.5 MPa and increased to about −1.2 MPa in the afternoon (Figure 3a). An insufficient protocol would thus lead to higher (artificial) embolism in the afternoon, while no difference in (native) PLC was to be expected with an optimized protocol. For the cultivars Red Delicious and Braeburn, the afternoon values of leaf water potential were in the range of xylem tension leading to first cavitation events (P12), while in Golden Delicious, P12 was significantly lower (−2.48 MPa; Beikircher et al. 2013). For the other cultivars, no information on vulnerability to drought-induced embolism is available, but many apple cultivars were reported to exhibit a P12 around −1 MPa (Jones et al. 1989, Nardini and Salleo 2000, Christensen-Dalsgaard and Tyree 2014). However, under transpiring conditions, leaf water potential is assumed to be lower (i.e., more negative) than the water potential in branches and stem, and thus, induction of embolism in the intact plants was unlikely. Accordingly, low native embolism was observed in all samples (Figure 3). Nevertheless, during midday, stomatal conductances were in the range of maximal stomatal conductance values measured in 2010 on Golden Delicious, Braeburn and Red Delicious (Beikircher et al. 2013), and thus, water columns in the xylem were under tension. Yet, we observed no differences in the level of native embolism with regard to time of day of harvesting (Figure 3a). Accordingly, also *k*_s_ and water contents of wood and bark did not differ between branches harvested in the afternoon and in the morning, respectively (Figure 3b–d).

**Figure 4.**
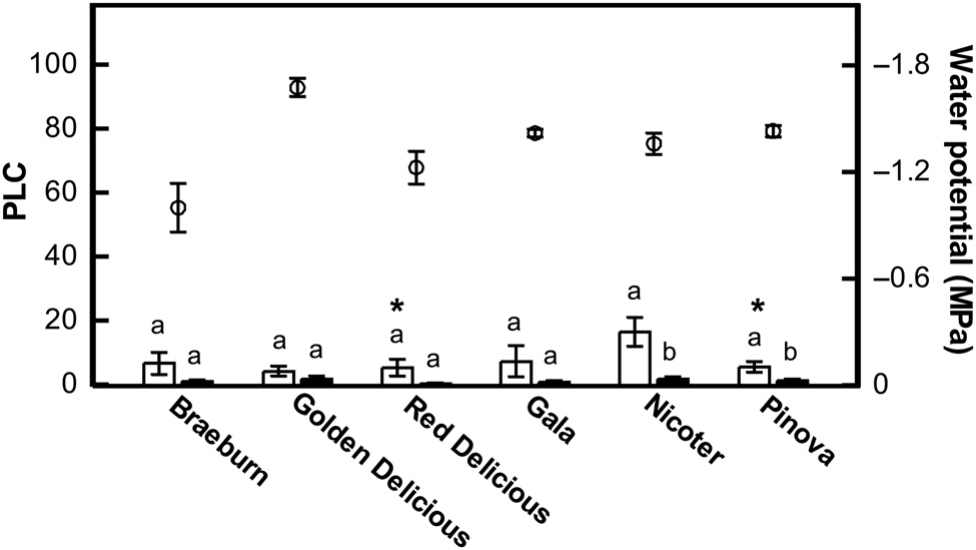
Percentage loss of hydraulic conductivity (PLC; bars) and leaf water potential (circles) of six different cultivars of *M. domestica*. Measurements were made on previous-year (open bars) and current-year (solid bars) shoots of branches harvested in July 2011. Letters show significant differences within a cultivar, and asterisks indicate significant differences from PLC in May (Figure 3). Means ± SE.

While no differences in PLC with regard to time of day of harvesting were observed, values significantly differed between shoot ages (Figure 3a). An artefact due to branch and conduit length (see above) can be excluded as previous-year samples were taken only ∼15 cm basal from current-year samples, and afternoon values did not differ from predawn values (Figure 3). Based on a second set of measurements in summer (Figure 4) as well as a follow-up study (data not shown), we assume that the higher amount in native embolism in previous-year shoots was related to winter embolism. As measurements were made in spring, restoration of the hydraulic system in previous-year shoots after winter may yet not have been completed. Furthermore, a certain amount of conduits can remain embolized in the following season (e.g., Christensen-Dalsgaard and Tyree 2014). In line with this argument, PLC of previous-year shoots decreased from on average 30% in May to 5% in July (Figures 3 and 4).

According to the studies of Trifilo et al. (2014) and Venturas et al. (2015) though, rehydration for xylem tension relaxation prior to sampling may cause an opposite effect, i.e., artefactually decreased PLC due to refilling (see Introduction). For the following reasons, we do not think this is likely in our study species harvested and sampled according to our protocol. First, in the experiment to test for branch length (see above), embolism was only observed in short branches although they should more easily refill upon rehydration compared with long branches (Figure 2). Second, refilling in studied apple trees was never observed overnight although soils in the orchards were sufficiently supplied with water (soil water potential above −0.1 MPa; see Beikircher et al. 2013). As a matter of fact, depending on the cultivar, PLC ranged between 12 and 28% in previous-year shoots, regardless of time of day of harvesting (Figure 3a). Third, also the obvious seasonal course in native embolism with higher values in spring and low in summer (see above; Figure 4) does not indicate any artefactual refilling. The artefact of refilling upon xylem tension relaxation may be rather species specific (Trifilo et al. 2014, Venturas et al. 2015) and probably not of relevance in *Malus*. Also in other species such as *Acer saccharum* Marshall (Sperry et al. 1988; soaking overnight) and *Vaccinium myrtillus* L. (Ganthaler and Mayr 2015; rehydration for 30 min), no refilling effect was observed. In any case, further work is required to figure out how likely this artefact is in different species.

With their studies, Wheeler et al. (2013) and Trifilo et al. (2014) have kicked off a strong discussion on the critical issue of collecting plant material for hydraulic measurements. Researchers, currently working in the field of plant hydraulics, are thus in the awkward position of deciding whether previous studies may be questionable (Sperry 2013) and appropriately designing harvesting and sampling protocols for future measurements. Based on the findings of this study, we deem the protocol presented in this article appropriate for the harvesting and sampling of *M. domestica* branches, even if done under transpiring conditions in air. We assume it may also be applied for other species with comparable xylem anatomy and refilling potential. In any case, test measurements to exclude possible harvesting and/or sampling artefacts may be helpful, especially when commencing studies with critical species (e.g., long-vesseled angiosperms), and we recommend carefully consideration of the following aspects: (i) harvested plant material should exceed the maximum conduit length by several times. (ii) The harvested plant (part) should be immediately wrapped in dark plastic bags together with wet paper towels and recut several times under water. This is of particular importance when the first cut has been made in air. (iii) The basal ends of branches should be kept in water for at least 30 min to relax xylem tension prior to packing up and transporting it to the laboratory. Girdling tests as proposed by Trifilo et al. (2014) may be carried out to exclude refilling in the respective species. Alternatively to rehydration, predawn harvesting or covering plants in dark plastic bags several hours prior to cutting may also provide a good opportunity to avoid xylem tensions during harvesting and sampling and allow to pass on the rehydration step. (iv) The target section should be excised under water by recutting the harvested plant part repeatedly (in centimetre-steps and at both ends alternatively) to final sample length. (v) Finally, the sample ends should be trimmed several times with a sharp razor blade or wood-carving knife, thereby avoiding squeezing of conduits. Like in the study presented, experiments comparing different branch sections and harvesting times might be useful to test harvesting and sampling protocols.

## Conflict of interest

None declared.

## Funding

This study was financed by the Austrian Science Fund (FWF; project L556-B16 ‘Winter damage on apple trees’ and project T667-B16 ‘Hydraulics of juvenile trees’) and is linked to the COST Action FP1106 ‘STReESS’. Funding to pay the Open Access publication charges for this article was provided by the Austrian Science Fund (FWF).

## References

[TPV130C1] Beikircher B, Mayr S (2008) The hydraulic architecture of *Juniperus communis* L. ssp. *communis*: shrubs and trees compared. Plant Cell Environ 31:1545–1556. 18657057 10.1111/j.1365-3040.2008.01860.x

[TPV130C2] Beikircher B, Mayr S (2013) Winter peridermal conductance of apple trees: lammas shoots and spring shoots compared. Trees 27:707–715. doi:10.1007/s00468-012-0826-0 23794789 10.1007/s00468-012-0826-0 PMC3688303

[TPV130C3] Beikircher B, De Cesare C, Mayr S (2013) Hydraulics of high-yield orchard trees: a case study of three *Malus domestica* cultivars. Tree Physiol 33:1296–1307. doi:10.1093/treephys/tpt096 24319028 10.1093/treephys/tpt096

[TPV130C4] Choat B, Jansen S, Brodribb TJ et al (2012) Global convergence in the vulnerability of forests to drought. Nature 491:752–756. 23172141 10.1038/nature11688

[TPV130C5] Christensen-Dalsgaard KK, Tyree MT (2014) Frost fatigue and spring recovery of xylem vessels in three diffuse-porous trees in situ. Plant Cell Environ 37:1074–1085. doi:10.1111/pce.12216 24117494 10.1111/pce.12216

[TPV130C6] Cochard H, Badel E, Herbette S, Delzon S, Choat B, Jansen S (2013) Methods for measuring plant vulnerability to cavitation: a critical review. J Exp Bot 64:4779–4791. doi:10.1093/jxb/ert193 23888067 10.1093/jxb/ert193

[TPV130C7] Cohen S, Bennink J, Tyree M (2003) Air method measurements of apple vessel length distributions with improved apparatus and theory. J Exp Bot 54:1889–1897. doi:10.1093/jxb/erg202 12815034 10.1093/jxb/erg202

[TPV130C8] Dixon HH (ed.) (1914) Transpiration and the ascent of sap in plants. Macmillan and Co., London, UK, 216 p.

[TPV130C9] Ewers FW, Fisher JB (1989) Techniques for measuring vessel lengths and diameters in stems of woody plants. Am J Bot 76:645–656. doi:10.2307/2444412

[TPV130C10] Ganthaler A, Mayr S (2015) Dwarf shrub hydraulics: two Vaccinium species (*Vaccinium myrtillus, Vaccinium vitis-idaea*) of the European Alps compared. Physiol Plant 155:424–434. 25677081 10.1111/ppl.12333 PMC4949559

[TPV130C11] Jones HG, Higgs KH, Bergamini A (1989) The use of ultrasonic detectors for water stress determination in fruit trees. Ann Sci For 46:338s–341s. doi:10.1051/forest:19890575

[TPV130C12] Melcher PJ, Holbrook NM, Burns MJ, Zwieniecki MA, Cobb AR, Brodribb TJ, Choat B, Sack L (2012) Measurements of stem xylem hydraulic conductivity in the laboratory and field. Methods Ecol Evol 3:685–694. doi:10.1111/j.2041-210X.2012.00204.x

[TPV130C13] Nardini A, Salleo S (2000) Limitation of stomatal conductance by hydraulic traits: sensing or preventing xylem cavitation? Trees 15:14–24. doi:10.1007/s004680000071

[TPV130C14] Nolf M, Pagitz K, Mayr S (2014) Physiological acclimation to drought stress in *Solidago canadensis*. Physiol Plant 150:529–539. doi:10.1111/ppl.12100 24024793 10.1111/ppl.12100

[TPV130C15] Sperry J (2013) Cutting-edge research or cutting-edge artefact? An overdue control experiment complicates the xylem refilling story. Plant Cell Environ 36:1916–1918. 23763611 10.1111/pce.12148

[TPV130C16] Sperry JS, Donnelly JR, Tyree MT (1988) A method for measuring hydraulic conductivity and embolism in xylem. Plant Cell Environ 11:35–40. doi:10.1111/j.1365-3040.1988.tb01774.x

[TPV130C17] Torres-Ruiz JM, Jansen S, Choat B et al (2015) Direct X-ray microtomography observation confirms the induction of embolism upon xylem cutting under tension. Plant Physiol 167:40–43. doi:10.1104/pp.114.249706 25378693 10.1104/pp.114.249706 PMC4281005

[TPV130C18] Trifilo P, Raimondo F, Lo Gullo MA, Barbera PM, Salleo S, Nardini A (2014) Relax and refill: xylem rehydration prior to hydraulic measurements favours embolism repair in stems and generates artificially low PLC values. Plant Cell Environ 37:2491–2499. doi:10.1111/pce.12313 24588546 10.1111/pce.12313

[TPV130C19] Tyree MT, Sperry JS (1988) Do woody plants operate near the point of catastrophic xylem dysfunction caused by dynamic water stress? Answers from a model. Plant Physiol 88:574–580. doi:10.1104/pp.88.3.574 16666351 10.1104/pp.88.3.574 PMC1055627

[TPV130C20] Tyree MT, Zimmermann MH (eds) (2002) Xylem structure and the ascent of sap, 2nd edn Springer, Berlin, 283 p.

[TPV130C21] Tyree MT, Davis SD, Cochard H (1994) Biophysical perspectives of xylem evolution: is there a tradeoff of hydraulic efficiency for vulnerability to dysfunction? IAWA J 15:335–360. doi:10.1163/22941932-90001369

[TPV130C22] Venturas MD, MacKinnon ED, Jacobsen AL, Pratt RB (2015) Excising stem samples underwater at native tension does not induce xylem cavitation. Plant Cell Environ 38:1060–1068. doi:10.1111/pce.12461 25292257 10.1111/pce.12461

[TPV130C23] Wheeler JK, Huggett BA, Tofte AN, Rockwell FE, Holbrook NM (2013) Cutting xylem under tension or supersaturated with gas can generate PLC and the appearance of rapid recovery from embolism. Plant Cell Environ 36:1938–1949. 23701011 10.1111/pce.12139

[TPV130C24] Zimmermann MH (1978) Hydraulic architecture of some diffuse-porous trees. Can J Bot 56:2286–2295. doi:10.1139/b78-274

